# Synergistic Immuno Photothermal Nanotherapy (SYMPHONY) for the Treatment of Unresectable and Metastatic Cancers

**DOI:** 10.1038/s41598-017-09116-1

**Published:** 2017-08-17

**Authors:** Yang Liu, Paolo Maccarini, Gregory M. Palmer, Wiguins Etienne, Yulin Zhao, Chen-Ting Lee, Xiumei Ma, Brant A. Inman, Tuan Vo-Dinh

**Affiliations:** 1 Department of Biomedical Engineering, Duke University, Durham, NC 27708 USA; 2 Department of Chemistry, Duke University, Durham, NC 27708 USA; 3 Department of Radiation Oncology, Duke University Medical Center, Durham, NC 27710 USA; 4 Department of Surgery, Duke University Medical Center, Durham, NC 27710 USA; 5 Fitzpatrick Institute of Photonics, Duke University, Durham, NC 27708 USA

## Abstract

Metastatic spread is the mechanism in more than 90 percent of cancer deaths and current therapeutic options, such as systemic chemotherapy, are often ineffective. Here we provide a proof of principle for a novel two-pronged modality referred to as Synergistic Immuno Photothermal Nanotherapy (SYMPHONY) having the potential to safely eradicate both primary tumors and distant metastatic foci. Using a combination of immune-checkpoint inhibition and plasmonic gold nanostar (GNS)–mediated photothermal therapy, we were able to achieve complete eradication of primary treated tumors and distant untreated tumors in some mice implanted with the MB49 bladder cancer cells. Delayed rechallenge with MB49 cancer cells injection in mice that appeared cured by SYMPHONY did not lead to new tumor formation after 60 days observation, indicating that SYMPHONY treatment induced effective long-lasting immunity against MB49 cancer cells.

## Introduction

Cancer is a severe threat to human life and results in more than 7 million deaths each year^1^. Many cancers exploit immune checkpoints to evade the anti-cancer immune response. Immune checkpoint inhibition is a promising immunotherapy that aims to reverse signals from immunosuppressive tumor microenvironments^2^. Programmed death-ligand 1 (PD-L1), a protein overexpressed by many cancers, contributes to the suppression of the immune system and cancer immune evasion. PD-L1 binds to its receptor, PD-1 found on activated T cells, and inhibits cytotoxic T-cell function, thus escaping the immune response^3^. To reverse tumor-mediated immunosuppression, therapeutic anti-PD-1/PD-L1 antibodies have been designed to block the PD-L1/PD-1 interaction. It has been demonstrated that PD-L1 blockade represents a promising therapeutic strategy using animal models of cancer^3, 4^.

The immune system response is also heightened by several temperature-induced mechanisms, such as antigen delivery by heat shock proteins (HSPs) and improved migration of lymphocytes to hot spots^5^. Immunotherapies can thus synergistically benefit from targeted thermal therapies, especially if mild hyperthermia (HT) is combined with precise thermal ablation of cancer cells^6^. As one of the first effective systemic cancer treatments, hyperthermia aims to increase tumor temperature above the normal value (~36 °C) to trigger local and systemic antitumor effects and/or ablate cancer cells. While HT at high temperature (>55 °C) can actually induce immediate thermal death (ablation) to targeted tumors, mild HT (fever-range <43 °C) can be used to improve drug delivery to tumors, improve cancer cell sensitivity to other therapies, and trigger potent systemic anti-cancer immune responses^7–10^. Traditional HT modalities such as microwaves, radiofrequency and ultrasound can control macroscopic heating around the tumor region, but cannot precisely target or ablate cancer cells in a timely manner. Conversely, nanoparticle (NP)-mediated thermal therapy has recently demonstrated the potential to combine the advantages of precise cancer cell ablation^11^ with benefits of mild HT in tumor microenvironments. NPs have a natural propensity to extravasate from the tumor vascular network and accumulate in and around cancer cells due to the enhanced permeability and retention (EPR) effect^12^. Among various types of nanoparticles, gold nanostars (GNS), whose sharp branches create a “lightning rod” effect that dramatically enhances the local electromagnetic (EM) field, are the most effective in converting light into heat for photothermal therapy (PTT)^13, 14^. The unique tip-enhanced plasmonics property of GNS can be optimally tuned in the near infrared (NIR) tissue optical window, where photons can travel further in healthy tissue to be ‘captured’ and converted into heat by GNS taken up preferentially in cancer cells^15–17^. We have investigated the PEGylated GNS biodistribution in mice as well as GNS uptake at both macroscopic and microscopic scales by using radiolabeling, CT and optical imaging methods^15^. In addition, a recent toxicity study of aptamer-loaded GNS found no signs of acute toxicity^18^. Even for a high dose of GNS at 48 mg/kg, no morphology changes in hepatocytes were observed and the GNS dose was well tolerated.

## Results and Discussion

**Figure 1:**
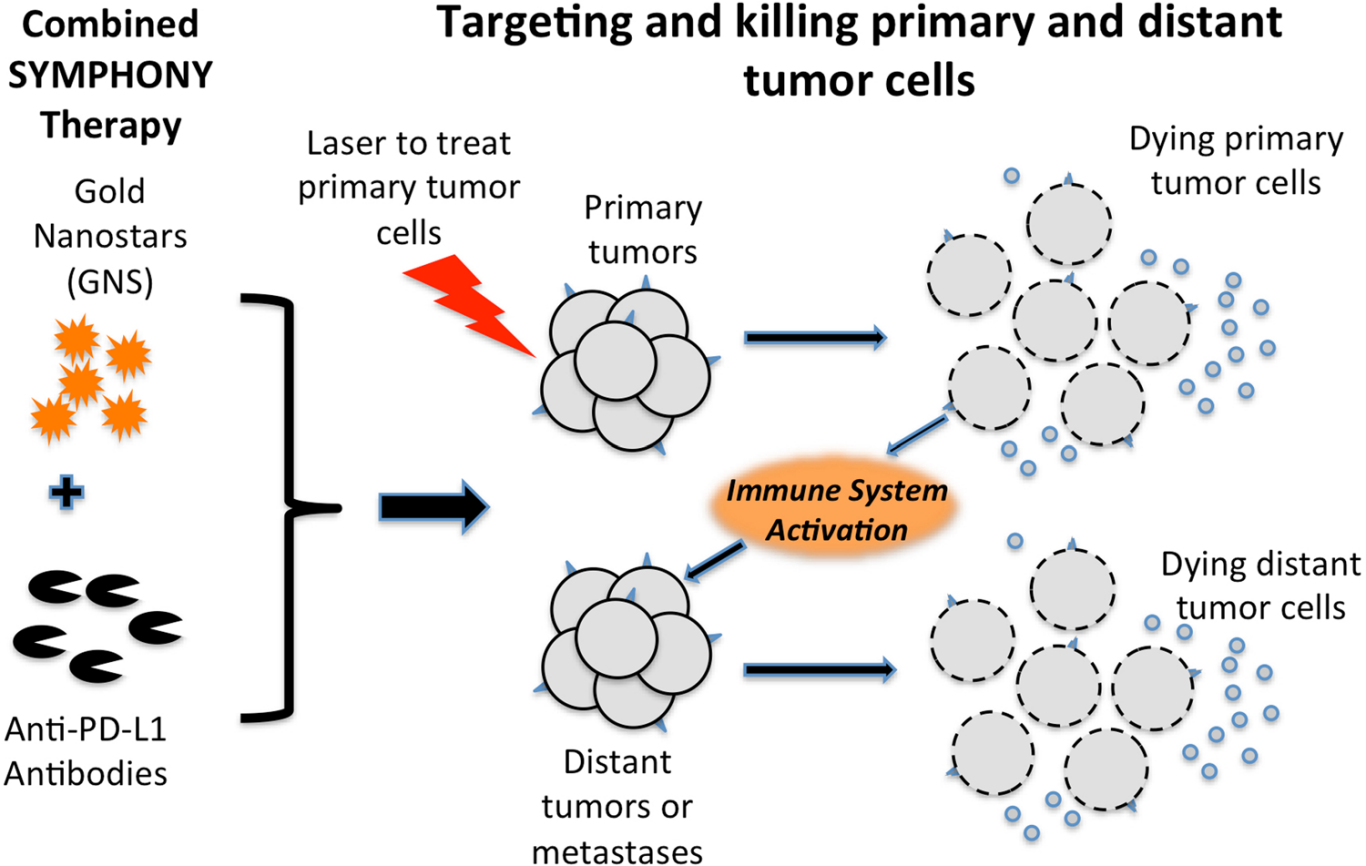
SYMPHONY: Synergistic Immuno Photothermal Nanotherapy. By disabling the tumor immune resistance using anti-PD-L1 antibodies and simultaneously ablating individual cancer cells using GNS-enabled photothermal therapy, SYMPHONY can trigger a powerful thermally enhanced systemic immune activation to rapidly eradicate locally aggressive as well as distant metastatic cancer.

Figure 1 schematically depicts the SYMPHONY concept, which combines anti-PD-L1 immunotherapy with GNS-mediated photothermal therapy for a two-pronged treatment modality. One therapeutic arm uses laser light to irradiate the primary tumor area where GNS have accumulated, resulting in generation of heat, which kills the primary tumor cells. Not only is there an immediate killing effect at the site treated with light, but this treatment also results in a general activation of the immune system, as evidenced by the fact that distant tumors not treated with light also show cancer cell killing. The second therapeutic arm involves administration of PD-L1 immune checkpoint blockade to disable cancer resistance. By suppressing the tumor defense, the tumor cells are now vulnerable to the killing action of immune cells that have been activated against the tumor by the nanoparticle photothermal therapy.

**Figure 2:**
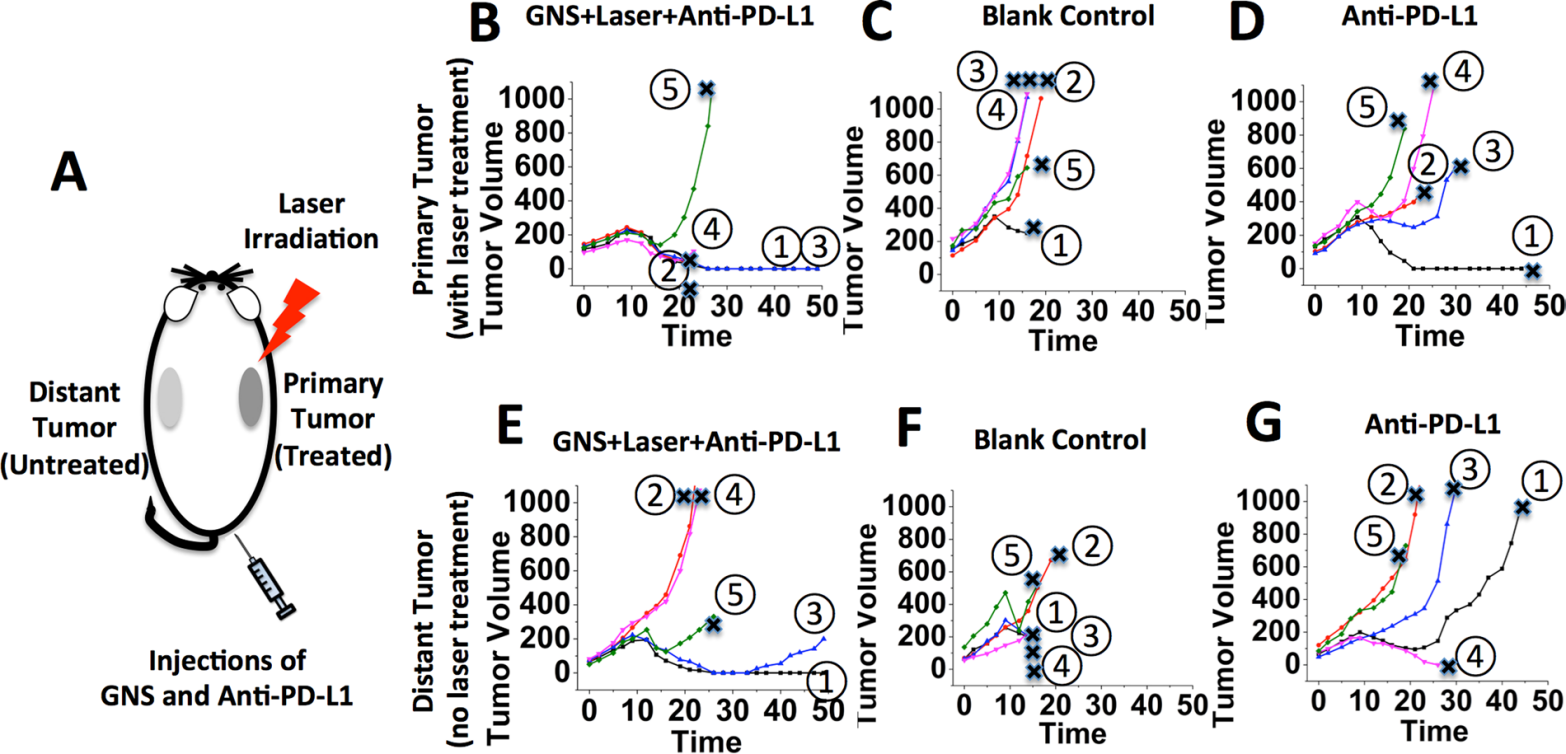
LEFT: (**A**) SYMPHONY treatment principle. The right flank tumor was treated photothermally and the left flank tumor not. RIGHT Top: Tumor growth for primary tumors in the SYMPHONY group (GNS + Laser + Anti-PD-L1) (**B**), in the control group (**C**), and in the group with anti-PD-L1 alone (**D**). RIGHT Bottom: Tumor growth for distant tumors in the SYMPHONY group (GNS + Laser + Anti-PD-L1) (**E**), in the control group (**F**), and in the group with anti-PD-L1 alone (**G**). The laser irradiation of photothermal therapy was performed only on the primary tumors. The line stopped (×sign in black color) if the mouse was sacrificed. The tumor volume unit is mm^3^ and the time unit is day.

Laboratory animal studies were performed using C57BL/6 mice implanted with MB49 bladder cancer cells to produce tumors on the right flank (‘primary’ tumor) and left flank (‘distant’ tumor, serving as the metastatic cancer model). The experiment results in Fig. 2 demonstrate the effectiveness of the SYMPHONY treatment. Fig. 2A schematically depicts the treatment protocol of the two-pronged SYMPHONY modality, where the right flank tumor was treated photothermally and the left flank was not. Figure 2B shows that the primary tumors treated with SYMPHONY shrank starting on Day 8. Two mice (#1 and #3) exhibited no measurable tumors from day 26 until day 49. Two mice (#2 and #4) had primary tumor sizes that decreased, but they were sacrificed due to contralateral tumor growth. One mouse (#5) responded to treatment for 2 weeks then the tumor grew again. All primary tumors in the blank control group exhibited rapid growth until the mice were sacrificed (Fig. 2C). For the mice group with primary tumor receiving anti-PD-L1 antibody alone, there is only 1 mouse (#1) that has a primary tumor response and it was sacrificed due to progression of cancer on the contralateral side (Fig. 2D). It is noteworthy that the therapeutic response for primary tumors treated using SYMPHONY was significantly better than that with anti-PD-L1 therapy alone.

Figure 2E shows that of the contralateral tumors (used as the metastasis model system) that did not receive any laser treatment, two (#1 and #3) had completely disappeared with SYMPHONY treatment of the primary tumor, demonstrating important abscopal immune responses. Untreated contralateral tumors in the blank control group exhibited rapid and significant growth until the mice were sacrificed (Fig. 2F). For the group with distant tumors receiving anti-PD-L1 antibody alone, there is only 1 mouse (#4) that has a distant tumor response and it was sacrificed due to the large tumor growth on the other side (Fig. 2G). The SYMPHONY strategy shows clear improvement over anti-PD-L1 therapy alone for cancer treatment of both primary tumours (Fig. 2B and D) and distant metastasis (Fig. 2E and G).

The results in Fig. 2 demonstrate the immediate killing effect at the primary tumor site (Fig. 2B) as well as evidence of the immune system activation resulting in tumor responses on contralateral tumors that were not treated with phototherapy (Fig. 2E). The results in Fig. 2E show evidence of an abscopal effect with SYMPHONY. The abscopal effect occurs when distant untreated tumors regress during treatment of a primary tumor. The observed abscopal effect is thought to be due to immune activation and generally indicate the induction of effective immunity^19^. On the contrary, Fig. 2F shows that the tumors in the blank control group had rapid growth.

**Figure 3:**
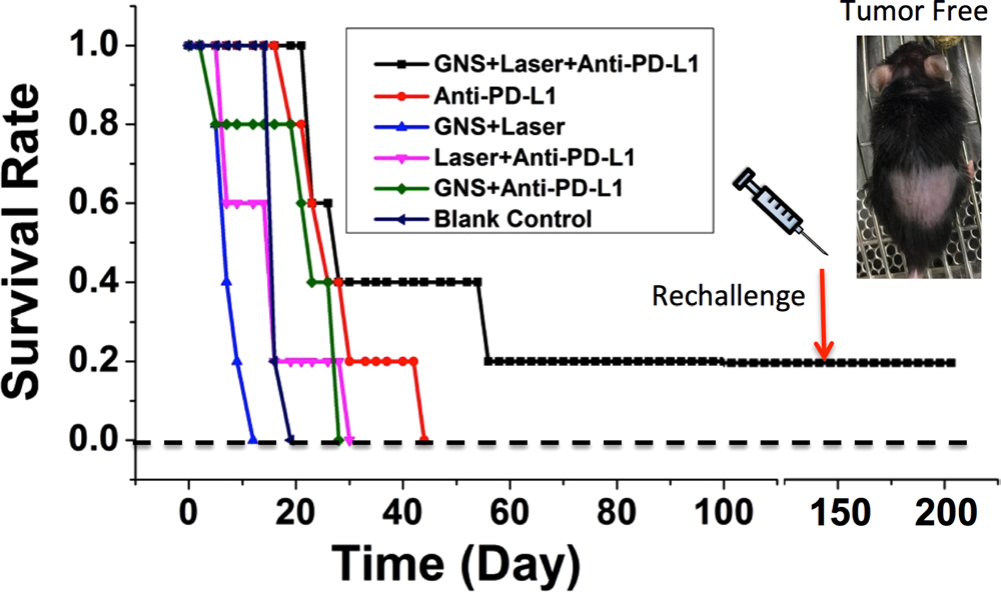
Kaplan-Meier (K-M) overall survival curve for aggressive bladder cancer murine models. GNS (2 mg per mouse) was intravenously (IV) injected through tail vein on Day 0 and laser treatment (808 nm, 0.6 W/cm^2^) was performed on Day 1. Anti-PD-L1 antibody was intraperitoneally (IP) injected every 3 days (200 μg per injection). Only SYMPHONY group (GNS + Laser + Anti-PD-L1) has 2 survival mice (40%) at the end of 49 days. As of today (>200 days) one mouse (20%) is deemed cured even after a rechallenge experiment on day 145. All control groups have no survival mouse on day 43.

Figure 3 depicts the Kaplan-Meier (K-M) overall survival curve, which shows survival improvement of the SYMPHONY treatment over anti-PD-L1 immunotherapy alone. At the end of 49 days, the survival rate for SYMPHONY group is 40% while it is 0% for all other groups including the anti-PD-L1 antibody monotherapy group. Anti-PD-L1 therapy alone, however, did show a modest therapeutic benefit (tumor growth delay) compared to untreated controls. For both primary and distant tumors (metastasis model), a combination of anti-PD-L1 therapy with photothermal nanotherapy showed strong synergism. The tumor-free mouse was monitored for more than 90 days and there was no tumor recurrence. We also performed rechallenge experiment in the tumor-free mouse by injecting 250,000 MB49 bladder cancer cells under the dorsal skin, and it did not lead to tumors within 60 days indicating that SYMPHONY had induced effective long-lasting immunity against MB49 (Fig. 3 inset). Additionally, we performed the SYMPHONY treatment by injecting GNS intratumorally on another group of 5 mice, resulting in one tumor-free mouse, which also remained tumor free after rechallenge. To date the two tumor-free mice, i.e., (one with IV injection (Fig. 3 inset) and the other with intratumoral injection of GNS (data not shown) treated with SYMPHONY survived up to 7 months with no tumor recurrence. The experiment result that no tumors grew after cancer re-challenge, shows a proof that SYMPHONY triggers a long-term cancer vaccine effect. The synergetic effect of the combination of GNS-enabled PTT and anti-PD-L1 treatment was analyzed with the Jin’s formula^20–22^. Using the survival rate as the therapeutic effect *E*, we evaluated SYMPHONY’s synergistic factor *Q*_*SYM*_ to demonstrate synergistic effect. The survival rates 30 or 40 days after treatment are the same and chosen for evaluation. The formula is ${Q}_{SYM}=\frac{{E}_{SYM}}{{E}_{PTT}+{E}_{IM}-{E}_{PTT}\times {E}_{IM}}$, where ${E}_{SYM}(=0.4)$, *E*_*PTT*_(=0), and *E*_*IM*_ (=0.2) are the survival rates of the combination SYMPHONY treatment, GNS-enabled PTT treatment only, and anti-PD-L1 immunotherapy treatment only, for 30 or 40 days after treatment. In the Jin’s method, Q < 0.85 shows antagonism, 0.85 ≤ Q < 1.15 shows additive effects, and Q ≥ 1.15 shows synergism. For the SYMPHONY treatment, the calculated *Q*_*SYM*_ is 2, which is larger than 1.15, indicating a synergistic effect. In addition, only the combination SYMPHONY treatment shows long-term survival and immunity after rechallenge. This observation further underlines the synergistic effect between photothermal therapy with gold nanostars and immunotherapy with anti-PD-L1 antibody.

**Figure 4:**
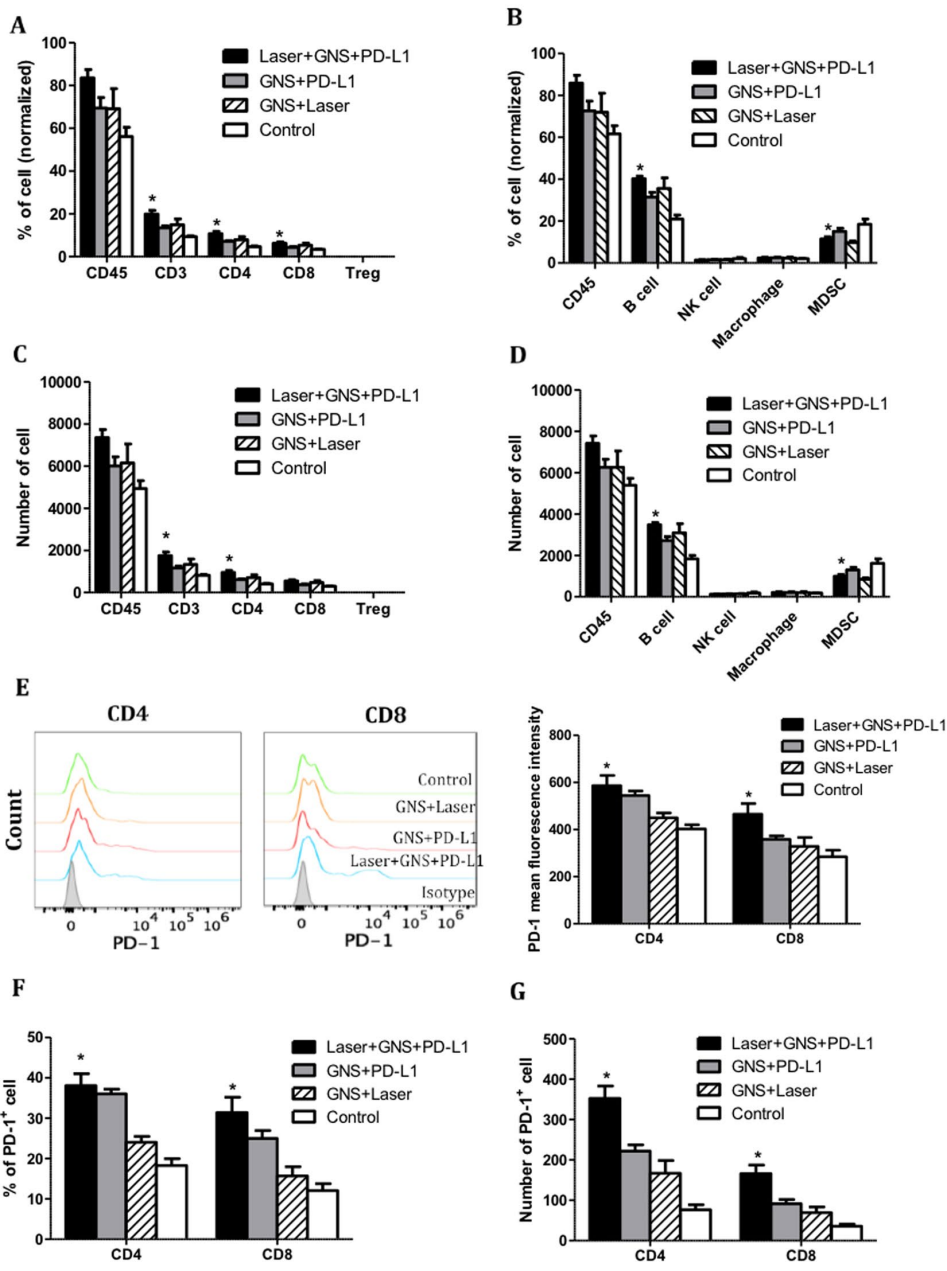
Immune cell phenotyping in the spleen. Single cell suspensions were processed from the spleen. (**A,B**) The percentage and (**C**,**D**) absolute cell number of total leukocytes (CD45), total T cells (CD3), CD4, CD8, T regulatory cells (CD4/CD25/FOXP3) and (B) B cells (CD19), NK cells (CD335), macrophages (CD11b/F4/80) and MDSC (CD11b/Gr1) were analyzed by flow cytometry. The percentage of cells was normalized to total single cells in each sample. (**E**) Mean fluorescence of PD-1 expression on CD4 and CD8 T cells. **(F**) The percentage and (**G**) absolute cell number of PD-1 + CD4 and PD-1 + CD8 T cells. *p < 0.05, compared to control group. One-way ANOVA analysis was performed.

To study the effects of SYMPHONY treatment on anti-tumor immune response, spleens were collected from a separate cohort (5 mice per group) of MB49-tumor bearing mice 7 days post treatment for immune cell phenotyping. In spleens, we found that SYMPHONY treatment significantly increased the percentage of total T cells, CD4, CD8 T cells and B cells (Fig. 4A and B). The absolute numbers of total T cells, CD4 T cells and B cells were also increased in the mice receiving the combination treatment (Fig. 4C and D). On the other hand, both the percentage and cell number of myeloid-derived suppressor cells (MDSC) were significantly reduced in the combination treatment group. We also investigated the roles of photothermal therapy and PD-L1 treatment on PD-1 expression on T cells. The results indicated that the combination treatment upregulated PD-1 expression on CD4 and CD8 T cells and also synergistically increased the percentage and cell number of PD-1^+^CD4 and PD-1^+^CD8 T cells (Fig. 4E and G).

In summary, our SYMPHONY experiment results provide a proof of principle for the synergistic effect from immune triggering of targeted photothermal therapy and anti-PD-L1 immunotherapy, and show great promise to manage not only accessible primary tumors but also cancer metastasis in future cancer treatment.

## Methods

Gold nanostars (GNS) were synthesized following a modified method from our toxic surfactant-free method^14^. In short, 12-nm gold sphere nanoparticles synthesized by reducing HAuCl_4_ with trisodium citrate were used as seeds, which were rapidly mixed with AgNO_3_, ascorbic acid and HAuCl_4_. The synthesized GNS were coated with polyethylene glycol (PEG, M.W. 6000) by gold-thiol bond to improve *in vivo* stability and circulation time. The PEG-functionalized GNS were concentrated to 20 mg/ml for injection. The gold mass concentration was measured with atomic absorption spectroscopy (AAS).

MB49 bladder cancer cells were subcutaneously implanted into C57BL/6 mice (250,000/100,000 cells in right/left flank, respectively). These tumors grew for 10–14 days to achieve a 50–150 mm^3^ volume (right flank tumor). At this point, animals were intravenously (IV) injected through tail vein with GNS at a dose of 2 mg in 100 µL of PBS solution (Day 0). One day after GNS injection (Day 1), the photothermal therapy treatment was performed with an 808-nm laser for 10 minutes at a power density of 0.6 W/cm^2^. During the treatment, mice were anesthetized with isoflurane delivered by nose cone. Half hour after laser irradiation, anti-PD-L1 antibody (200 µg per mouse) was intraperitoneally (IP) injected. The antibody was repeatedly injected every 3 days until the mice were sacrificed or tumors are completely cured. Tumor growth and body weight were monitored every two days. Humane endpoint euthanasia was performed if mice showed adverse reactions to treatments, lost greater than 15% of their body weight, the volume of single flank tumor was greater than 1,000 mm^3^ or the total volume of two flank tumors was larger than 1,500 mm^3^. All animal studies were approved by the Institutional Animal Care and Use Committee of Duke University (protocol number A114-15-04) and all methods were performed in accordance with guidelines and regulations.

For immune cell phenotyping, a separate cohort of MB49 tumor bearing animals were prepared and treated as above. In this case, spleens and tumors were harvested from MB49-tumor bearing mice 7 days post treatment. Tumor-infiltrating lymphocytes were isolated by dissociating tumor tissue using a gentleMacs dissociator (Miltenyi Biotec) in the presence of 0.2 mg/ml DNase I, 1 mg/ml collagenase IV and 0.1 mg/ml hyaluronidase (Sigma). The cells were incubated at 37 °C for 1 hour and then passed through a 40 μM strainer (Falcon) to get single cell suspensions. For flow cytometry, the cells were stained with anti-CD16/32 Ab (BioLegend) to block non-specific binding and LIVE/DEAD Fixable Violet Dead Cell Stain Kit (Thermo Fisher) to exclude dead cells. The cells were then stained with antibodies against CD45, CD3, CD4, CD8, CD25, CD19, CD335, CD11b, F4/80, Gr1 and PD-1 (BioLegend) and analyzed by multiparameter flow cytometry (FACSCanto, BD Bioscience). Intracellular staining for FOXP3 (BioLegend) was carried out using a Cytofix/Cytoperm, Fixation/Permeabilization Solution Kit according to the manufacturers’ instructions (BD Bioscience). Analysis of data was performed with FlowJo (Tree Star).
